# Variation and Variability in *Drosophila* Grooming Behavior

**DOI:** 10.3389/fnbeh.2021.769372

**Published:** 2022-01-11

**Authors:** Joshua M. Mueller, Neil Zhang, Jean M. Carlson, Julie H. Simpson

**Affiliations:** ^1^Interdepartmental Graduate Program in Dynamical Neuroscience, University of California, Santa Barbara, Santa Barbara, CA, United States; ^2^Department of Physics, University of California, Santa Barbara, Santa Barbara, CA, United States; ^3^Department of Molecular, Cellular, and Developmental Biology, University of California, Santa Barbara, Santa Barbara, CA, United States; ^4^Neuroscience Research Institute, University of California, Santa Barbara, Santa Barbara, CA, United States

**Keywords:** *Drosophila*, variability, variation, neural circuits, motor sequence, behavior

## Abstract

Behavioral differences can be observed between species or populations (variation) or between individuals in a genetically similar population (variability). Here, we investigate genetic differences as a possible source of variation and variability in *Drosophila* grooming. Grooming confers survival and social benefits. Grooming features of five *Drosophila* species exposed to a dust irritant were analyzed. Aspects of grooming behavior, such as anterior to posterior progression, were conserved between and within species. However, significant differences in activity levels, proportion of time spent in different cleaning movements, and grooming syntax were identified between species. All species tested showed individual variability in the order and duration of action sequences. Genetic diversity was not found to correlate with grooming variability within a species: *melanogaster* flies bred to increase or decrease genetic heterogeneity exhibited similar variability in grooming syntax. Individual flies observed on consecutive days also showed grooming sequence variability. Standardization of sensory input using optogenetics reduced but did not eliminate this variability. In aggregate, these data suggest that sequence variability may be a conserved feature of grooming behavior itself. These results also demonstrate that large genetic differences result in distinguishable grooming phenotypes (variation), but that genetic heterogeneity within a population does not necessarily correspond to an increase in the range of grooming behavior (variability).

## Introduction

Differences in phenotype arise from differences in genotype. Changes in DNA account for variation in traits among species, and differences between individuals of the same species. Animal behavior contains phenotypes partially under genetic control, and specific genes associated with observable differences in behavior between and within species have been uncovered (Baker et al., 2001; Johanssen, 2014). Different mouse species exhibit variation in monogamy and parental care, and different fly species show variation in courtship song, food preference, and larval digging (Bendesky et al., 2017; Kim et al., 2017; Ding et al., 2019; Markow, 2019; Auer et al., 2020). From endangered species to agricultural crops to virus variants, genetic diversity affects organismal success. Within a species, natural variations in DNA sequences produce individual mice that differ in aggression and or flies that implement different foraging strategies (Anderson, 2016; Allen et al., 2017) and advantageous variants can be selected. Mutant screens have also uncovered gene variants associated with differences in locomotion, courtship routines, and sleep patterns, among other complex behaviors (Baker et al., 2001; Sokolowski, 2001; Ayroles et al., 2015; Gaertner et al., 2015).

Some behaviors can be performed in different ways even by genetically identical organisms or by the same individual in repeated trials. Behavioral variability can be advantageous as a bet-hedging strategy against unstable environmental conditions (Kain et al., 2015). Phenotypic variability in behaviors ranging from birdsong to escape trajectories can increase individual success, but also fitness in a population, suggesting that variability itself can be a selectable trait. Experiments in *Drosophila melanogaster* demonstrate that the degree of behavioral variability in locomotion is partially controlled by genetic expression of proteins such as teneurin-α, a cell adhesion molecule (Honegger and de Bivort, 2018). Additionally, silencing a subset of neurons in the central complex modifies the degree of variability of locomotor behavior (Honegger and de Bivort, 2018). Differences in neurodevelopment and synaptic connectivity can also result in behavioral variability (Linneweber et al., 2020). Together, these observations suggest that factors at both at the population (genetic) and individual (neuronal) levels contribute to behavioral variability.

*Drosophila* grooming shows behavioral variability. Fruit flies live in dirty environments, from laboratory vials to rotting fruit, and they perform grooming actions to remove accumulated particulates. Grooming has been observed in several *drosophilid* species and is important for survival (Szebenyi, 1969; Spruijt et al., 1992; Zhukovskaya et al., 2013). Past work demonstrated that the leg movements used in grooming are stereotyped, but the sequences of actions are flexible as opposed to fixed. While the rules underlying grooming do exhibit observable structure in flies (Seeds et al., 2014; Mueller et al., 2019) and in mice (Fentress and Stilwell, 1973; Geuther et al., 2021), different sensory experiences and life histories may influence grooming behavior. These results lead us to ask: how much of variability in fruit fly grooming is under genetic control?

**FIGURE 1:**
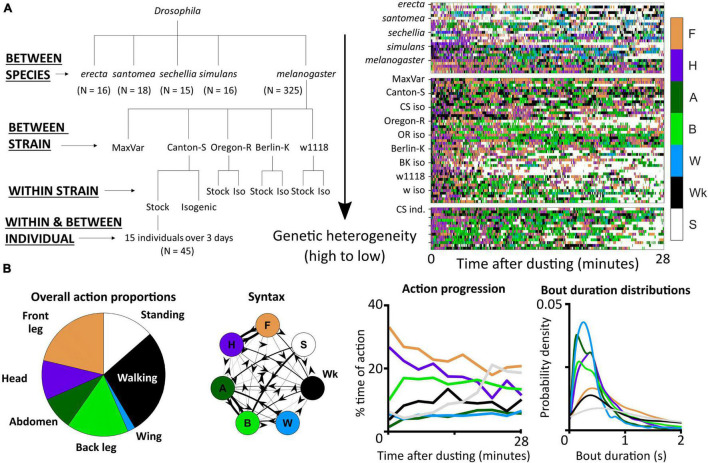
Grooming variability dataset and analysis overview. **(A)** In total, *N* = 390 male flies were dusted and their activity was recorded for approximately half an hour each. Five *drosophilid* species, four *melanogaster* stock lines, one interbred *melanogaster* line, and six isogenic *melanogaster* lines were analyzed for similarity, differences, and stereotypy in grooming and non-grooming behaviors. On the left is a schematic of the different *drosophilid* groups included in this analysis. Higher levels of the tree indicate higher levels of genetic diversity (scale is relative, not absolute). On the right is a sample of ethograms generated by automated annotation of video. Color indicates the occurrence of the five grooming actions (F, front leg cleaning; H, head grooming; A, abdomen grooming; B, back leg cleaning; W, wing grooming) and two non-grooming actions (Wk, walking; S, standing). **(B)** Features scored from ethograms provide summary representations of behavior. Shown here are sample visualizations of behavioral metrics analyzed in this work. On the left, the proportion of time spent in different actions provides the coarsest description of the behavioral response to a dust stimulus. Regardless of genotype, all flies exhibit variable (not fixed) action sequences consisting of the same set of five grooming actions, walking, and standing after exposure to irritant. Next, action transition probabilities (syntax) describe the likelihood of performing consecutive actions. Arrow directions and thicknesses represent the probability of performing an action, given the identity of the previous action. Shown next is an example behavioral progression, which depicts the proportion of time spent in each action over a sliding window. Most flies follow a typical behavioral progression pattern: initial anterior grooming followed by increased posterior grooming. The amount and timing of walking and standing, however, can vary significantly between flies. Finally, action (bout) duration distributions describe the range of action lengths. All example features shown here are scored from Canton-S flies.

To address this question, we evaluated different features of dust-induced grooming behavior by comparing their values between groups and their range within groups. The raw behavioral data and features we examine here are schematized in Figure 1. In particular, we focus on the transition probabilities between actions that compose the grooming sequence, which we refer to as “syntax”; in linguistics, this term refers to the rules that indicate how words and phrases may be combined to form sentences, so it is borrowed here to indicate that action transitions also conform to rules.

First, we evaluated differences in phenotype between genetically-distinct populations, such as *drosophilid* species. Differences in grooming behavior when members of different species are compared can reasonably be attributed to differences in their DNA sequences.

In a wild and genetically diverse population of flies, there may be mutations that change grooming behavior, but lab strains of *Drosophila melanogaster* are largely clonal—all individuals should have the same genotype. We next compared the grooming behavior of different lab strains, and then of individuals within a given lab strain. We hypothesized that genetic heterogeneity might contribute to the magnitude of grooming variability. By interbreeding or isogenizing *melanogaster* lab strains, we generated stocks with high and low genetic diversity, but we find that all groups exhibited similar variability in measured grooming features. Intra-genotypic or phenotypic variability has also been observed in fly locomotor behavior, and some populations exhibit a wide range while others exhibit a narrower one (Ayroles et al., 2015).

Genetic differences among *drosophilid* species and strains may underlie variation in the syntax of their cleaning movements, but all flies show variability in the exact sequence of those movements. Furthermore, even the same fly tested on sequential days revealed sequence variability. The extent of within-fly differences in syntax were similar to between-fly differences: flies were no more similar to themselves over time than they were to other flies on a given day. Finally, flies stimulated using optogenetic manipulation to induce grooming exhibited increased stereotypy, but within-individual grooming variability between stimulation sessions was not fully abolished. These data show that genetic heterogeneity plays a limited role in the variability of grooming behavior, and that differences in sensory experience contribute but do not account for all observed variability. The widespread nature of grooming variability suggests that it may be an important feature, but our experiments indicate the need to search for alternative causes, perhaps including developmental stochasticity, differences in internal state, or noisy neural circuit dynamics.

## Results

In this work, *N* = 390 male flies were covered in dust and their grooming behavior was recorded for approximately 30 min each (Figure 1A). We analyzed flies from five *drosophilid* species (*melanogaster*, *santomea*, *sechellia*, *simulans*, and *erecta*), which are genetically distinct—separated by millions of years of evolution—and inhabit different ecological niches. We also examined four common *melanogaster* lab stocks (Canton-S, Oregon-R, Berlin-K, and w1118), and several isogenic lines derived from these parent stocks in our laboratory.

To analyze this large data set, we employed tools from computational ethology (Datta et al., 2019). An automated behavioral recognition system [ABRS, Ravbar et al. (2019)] was used to classify fly behavior into one of five grooming actions (front leg cleaning, head grooming, abdomen grooming, back leg cleaning, wing grooming) and two non-grooming actions (walking and standing). As a note, head grooming consists of actions that use the front legs to clean the antennae, eyes, and face, but sub-movements such as these were not easily detectable using the recording methodology employed here, so analysis was restricted to coarser spatiotemporal scales. After generating ethograms (behavioral time series records) for each fly, several grooming features were extracted (Figure 1B). We measure the average amount of time flies spend performing each of the grooming actions (plus standing and walking), the syntax of transition probabilities among these actions, the anterior-to-posterior progression, and the durations of bouts of grooming actions. We used classification analysis and various measures of stereotypy to quantify the variation (inter-species or inter-strain differences) and variability (intra-strain or intra-individual differences) of these characteristics.

### *Drosophilids* Exhibit a Robust Grooming Response but Different Syntax After Irritant Exposure

Across the *Drosophila* species tested here, five grooming actions were observed consistently, indicating a conserved behavioral response (Supplementary Figure 1). Previous work showed that these actions are sufficiently stereotyped to be reliably classified by manual and automated annotation in *melanogaster* (ABRS, see section “Materials and Methods”) (Mueller et al., 2019; Ravbar et al., 2019). Here, the ABRS classifier was validated on training data for each species, showing comparable accuracy (see section “Materials and Methods” and Supplementary Figure 22), indicating that the movement primitives that make up grooming are stereotyped. No novel species-specific grooming actions were detected. Although some fine-scale movement differences may occur among species, they are beyond the spatial and temporal resolution of the current video and unlikely to affect the analysis of transition probabilities presented here. Analysis of mouse behavior indicates that grooming subroutines are largely stereotyped at high temporal resolution, increasing our confidence in this approach (Wiltschko et al., 2015).

**FIGURE 2:**
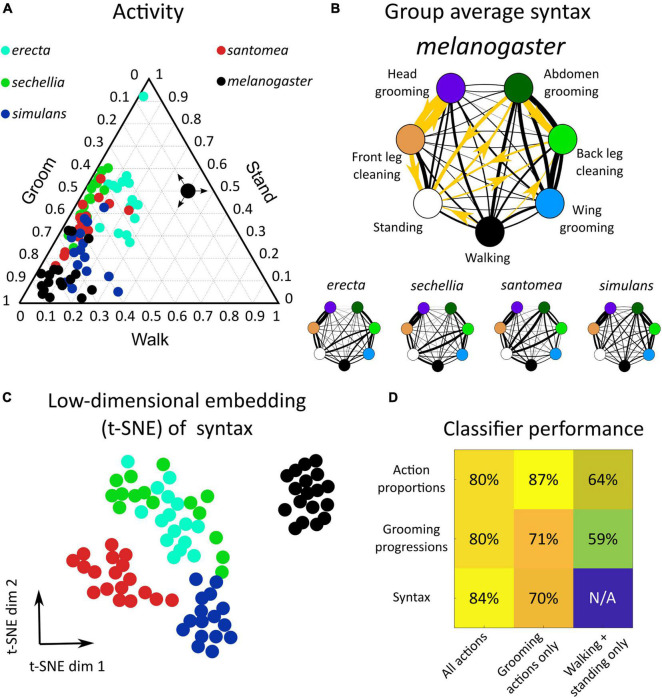
*Drosophila* species share behavioral features but exhibit between-species variation in action proportions and syntax in response to dust stimulus. **(A)** Dusting elicits a conserved behavioral response across *drosophilids*. Shown is a ternary plot of activity proportions for each species examined here (*N* = 65 flies total). Colored points represent a single fly, with color indicating species. The large black point with arrows indicates how to read activity proportions; the example point corresponds to 10% grooming, 40% walking, and 50% standing. **(B)** *Drosophilid* species produce a probabilistic behavioral sequence (as shown in Figure 1A), which can be characterized by the transition probabilities (syntax) between actions [as represented in Figure 1B, calculated as in Mueller et al. (2019)]. The mean syntax for each species is depicted as a graph, with nodes representing actions and edges indicating transition probability. Thicker edges indicate higher probabilities. On the *melanogaster* syntax graph, the 10 action transitions exhibiting the largest magnitude differences between *melanogaster* and non-*melanogaster* species are highlighted in gold. These differences are identifiable in anterior motif transitions, which use the front legs to perform grooming actions. Species also differ in their transitions between posterior grooming actions and non-grooming actions (walking and standing) **(C)** Each fly’s 42-dimensional syntax vector was plotted in two dimensions after dimensionality reduction using t-SNE. t-SNE preserves local distance structure, indicating that tightly grouped clusters of points are similar to one another. In this case, dimensionality reduction reveals that *drosophilid* species exhibit significant differences in syntax, as syntax vectors congregate by color. **(D)** Classification analysis confirms the qualitative clustering observed in C. Shown is a heat map of accuracy rates of 5-possibility multinomial logistic regression classifiers trained on behavioral features. For these samples, classification at chance would be 20%. Consistent classification accuracy values >20% indicate that species are highly separable by behavioral features. Simple features, such as behavioral proportions and progressions, classify individuals by species with high accuracy when grooming actions are included. Classification using only non-grooming actions (walking and standing) still yields classification above chance, indicating that species differ significantly in their overall activity levels. Syntax also allows for accurate classification, particularly when all action transitions are considered.

To quantify the behavioral response to dusting, the proportion of time spent grooming (as opposed to walking and standing) was calculated for each fly (Figure 2A). The proportions of total grooming time between species were all statistically different (Wilcoxon rank-sum test, *p* < 0.05, multiple comparison correction *via* Holm’s method; Supplementary Figure 23), but all species spent at least 35% of the time grooming, on average. In this analysis, a single stock line (Canton-S, *N* = 18) was used as the representative *melanogaster* group. Full action proportion distributions are shown in Supplementary Figure 2.

Behavior was then examined in more detail by considering all seven actions (five grooming movements plus walking and standing) and the progression of those actions over time, as in Figure 1B. These species exhibited a qualitatively similar behavioral progression, characterized by initial anterior grooming followed by increased posterior grooming, walking, and standing, but the relative proportions and timing of these behaviors over time differed (Supplementary Figure 3).

Syntax (the transition probabilities between discrete behaviors) was calculated from the ethogram of each dusted fly (*N* = 390). With seven behavioral states, 42 transitions were possible, excluding self-transitions. Thus, syntax was represented as a 42-dimensional vector for subsequent classification analysis and visualization. Syntax across all flies exhibited high transition probabilities within the anterior grooming motif (front leg cleaning, head grooming) and posterior grooming motif (abdomen grooming, back leg cleaning, wing grooming). The average syntax for each species is illustrated as a weighted, directed graph in Figure 2B.

Finally, continuous grooming action duration distributions (e.g., the distribution of how long each head grooming action was) were calculated from ethograms. Distributions of action durations were qualitatively similar across species and had probability peaks between 500 and 750 ms (Supplementary Figure 4). When considering the same action, no action duration distributions differed significantly in any pairwise comparison (e.g., comparing head grooming between *erecta* and *santomea*) between any species (two-way Kolmogorov-Smirnov test, no *p* values < 0.05 after correcting for multiple comparisons using Holm’s method).

Several significant differences in behavioral features between *melanogaster* and non-*melanogaster* species were identified. Supplementary Figure 5 illustrates differences in overall action proportions, around 36% of which differed between species. To compare syntax, transition probability distributions for each action transition (e.g., head cleaning to front leg rubbing) were compared between species in a pairwise manner. 38 of 42 unique syntax elements (90.5%) were significantly different between at least two species (Wilcoxon rank-sum test, *p* < 0.05, multiple comparison correction *via* Holm’s method). Overall, 125 of 420 (30%) of pairwise syntax comparisons revealed differences between species (see Supplementary Figure 24 for all *p* values).

Of these syntactic differences, 71 (60%) occurred between *melanogaster* and non-*melanogaster* species. In particular, posterior motif grooming transitions (transitions between abdomen grooming, back leg rubbing, and wing grooming) were consistently significantly different, on average, as were transitions between back leg rubbing, standing, and walking. Figure 2B illustrates these syntactic differences.

Figure 2C depicts a low-dimensional embedding of species syntax using t-SNE. This visualization suggests that different species possess distinguishable syntax, as points are aggregated by species. Low-dimensional visualizations of all behavioral features are illustrated in Supplementary Figures 5, 6.

Classification analysis was applied to behavioral features to verify this interpretation and quantify the degree of variation between species. Multinomial logistic regression classified flies by species according to behavioral proportions, progressions, and syntax with >80% accuracy (Figure 2D). Notably, classification was also possible with accuracy significantly above chance when only considering the proportions and progressions of non-grooming actions, walking and standing, indicating that species also vary in their overall activity levels.

Finally, entropy rates were calculated from syntax transition probabilities to quantify the degree of stereotypy in behavior. An entropy rate of zero would indicate complete stereotypy and perfectly predictable, repeated action sequences, while in this calculation, an entropy rate of one indicates an approximate 37% probability of correctly predicting the next action in a sequence (see section “Materials and Methods”). Supplementary Figure 7 shows that all species possess average entropy rates between zero and one, demonstrating that grooming sequences are neither fixed nor truly random. *melanogaster* flies possessed the lowest entropy (highest degree of stereotypy) due to high transition probabilities between head cleaning and front leg rubbing (Figure 2B). In summary, *drosophilid* species exhibit variation in grooming behavior—visible in proportion of time spent on different actions and the transition probabilities among them—but they all share common cleaning movements and variability in their exact action sequences.

### *Drosophila melanogaster* Strains Exhibit Variation in Grooming Behavior

Next, standard *Drosophila melanogaster* lab strains (Canton-S, Berlin-K, Oregon-R, w1118) were analyzed for differences in grooming features (full ethograms shown in Supplementary Figure 8). Behavioral proportions, progressions, and syntax differed between stocks, allowing for classification moderately above chance levels. Comparisons of grooming features can be found in Supplementary Figures 9–12.

**FIGURE 3:**
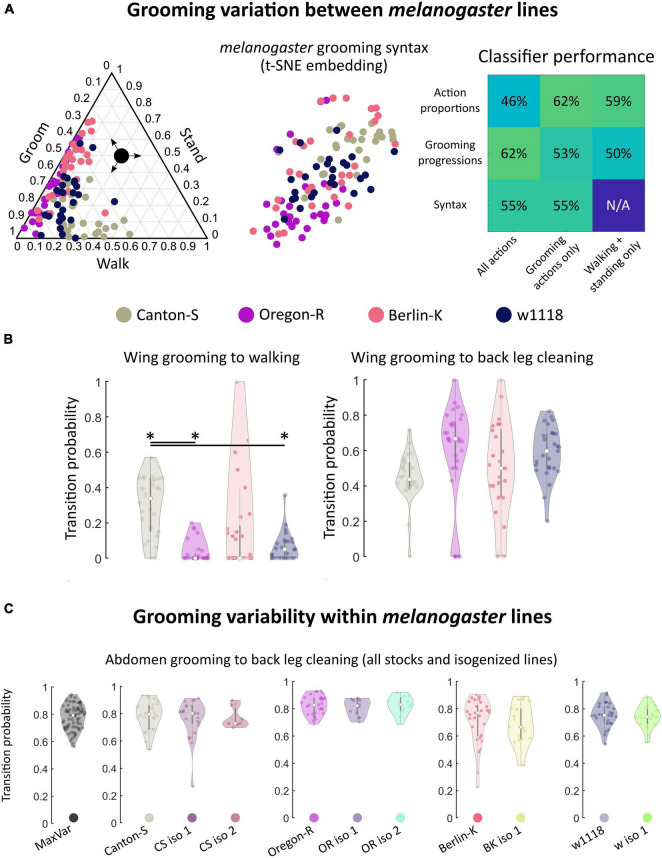
Within *melanogaster*, different stocks differences in syntax activity levels. Genetic homogeneity does not correspond to behavioral stereotypy. **(A)** *melanogaster* stocks (*N* = 111 flies total) exhibited variation in grooming syntax, though many features were shared. On the left is a ternary plot of grooming, walking, and standing proportions for each stock, similar to Figure 2A. Colored points represent individual flies. Shown in the middle is a t-SNE plot of syntax vectors, as in Figure 2C. The high degree of overlap in both of these plots illustrates that behavioral responses are qualitatively similar between some individuals of different stock lines. Classifier performance (similar to that shown in Figure 2D) is shown on the right. For these data, classification at chance is 25%. Performance above chance is still possible for stock lines. Classification performs similarly well for behavioral features regardless of their complexity; using just walking and standing behavioral proportions provides similar discriminability as using the full syntax. **(B)** Most syntax elements were similar between *melanogaster* stocks, but Canton-S flies walked more than other stocks. Due to differences in activity levels, some walking-related syntax elements differed between Canton-S flies and other stocks. Of the significantly different transitions, only two were within-motif transitions while the rest consisted mostly of transitions to and from walking and standing (Supplementary Figure 12). Shown on the left are the wing grooming to walking transition probability distributions for each *melanogaster* stock line. Significant differences in these distributions were observed between lines. On the right, distributions for a posterior grooming transition are shown; the vast majority of action transition distributions did not differ due to their large variances. **(C)** Variances of action transition distributions for stock lines, lines bred for maximum genetic heterogeneity (MaxVar), and lines bred to minimize genetic heterogeneity (iso) were compared (*N* = 252 total). Genetic homogeneity did not correspond to behavioral variability. Shown as an example are the distributions of abdomen grooming to back leg cleaning transitions. MaxVar flies did not exhibit a higher degree of variability (as measured by the variance of transition distributions) than stock lines. Isogenized lines did not exhibit a lower degree of variability than their parent stocks. *significantly different at *p* < 0.05.

Overall the proportion of time grooming could account for most of the differences observed between stocks. Figure 3A shows a ternary plot of activity, showing that Canton-S flies spend more time walking than other stocks. A t-SNE embedding of the syntax of *melanogaster* stocks is depicted in Figure 3A. Similar to the species analysis, all action transition probability distributions were compared in a pairwise manner to look for variation in syntax.

Only 19 of 42 unique syntax element comparisons (45%) differed significantly between any two stocks and, of these, only two within-motif transition (both posterior) differed significantly. Within-motif syntax elements are of particular interest because they represent the most common, most highly stereotyped action transitions observed across flies of all genotypes (see Figure 2B for visualizations of these transitions). The syntax element exhibiting the greatest statistically significant difference was the wing grooming to walking transition, shown in Figure 3B. The other significantly different transitions also mostly involved transitions to and from walking and standing, perhaps reflecting differences in overall activity levels (Supplementary Figure 12).

Classification accuracy was moderate but above chance for all features examined; as expected, variation within *melanogaster* was less pronounced than variation between species (compare Figures 2D, 3A). Variation within *melanogaster* stocks appears to be due to differences in overall activity levels, as classification using only non-grooming features (walking and standing proportions and progressions) yielded results similar to classification using full grooming behavior syntax. This is illustrated by the fact that Canton-S flies’ higher propensity to walk after grooming their wings is reflected both in their syntax and grooming proportions in Figure 3B.

Within Canton-S, activity levels separated male and female flies, as male flies tended to walk more than females (Supplementary Figure 13). Male and female flies also possessed somewhat different syntax; classification by syntax was 71%, where chance levels would be 50% for this comparison. This level of accuracy is higher than what was achievable when classifying *melanogaster* stock lines using syntax, but lower than the same comparison for interspecies data.

Since all flies examined showed variability in syntax, we wondered whether the extent of this variability differed among species or strains. Figure 3B also illustrates the high degree of variability in *melanogaster* syntax. The wing grooming to back leg cleaning transition exhibited the largest difference between median values of any syntax element (comparison of Canton-S and Oregon-R yielded this difference), but none of these distributions possessed detectable statistical differences due to their concomitantly large variances.

### Grooming Behavioral Variability Is Similar Across *melanogaster* Genotypes

To examine the potential relationship between genetic heterogeneity and behavioral variability, each *melanogaster* lab strain was compared to lines bred to maximize genetic heterogeneity (MaxVar) or minimize genetic heterogeneity (isogenic lines). These can be considered outbred and inbred strains. If variability in grooming syntax within a population is strongly related to genetic heterogeneity, we would expect populations with larger genetic heterogeneity to also contain flies with more variable syntax.

All lines, regardless of genetic heterogeneity, exhibit variable grooming (Supplementary Figure 14). To quantify variability, the variances of action transition probability distributions were calculated and compared. Only 6/252 (2.4%) transition probability distributions possessed statistically significantly different variances between MaxVar, Canton-S, and the isogenic lines out of all possible pairwise comparisons (Levene’s test, *p* < 05 after correction for multiple comparisons *via* Holm’s method). Moreover, none of these differences corresponded to within-motif transitions, indicating that variability of common transitions is similar regardless of genetic heterogeneity in a population. These findings also held for Oregon-R (8/252), Berlin-K (19/108), and w1118 (2/108) stock and isogenic comparisons. See Supplementary Figure 25 for all *p* values of pairwise action transition distribution variance comparisons.

Figure 3C provides the transition probability distributions for the most common posterior motif transition (abdomen grooming to back leg cleaning) for all stocks and stock-derived isogenic lines. This transition exhibits wide variability in many populations and even populations with smaller variability (CS iso 2) are not different enough to achieve statistical significance after accounting for multiple hypothesis testing.

We also examined stock lines derived from selected wild isolates (Mackay et al., 2012) to determine if these showed more or less grooming variability, as measured by syntax element variance values and Markov entropy. Their variability is comparable to that within lab stocks (Supplementary Figure 15).

**FIGURE 4:**
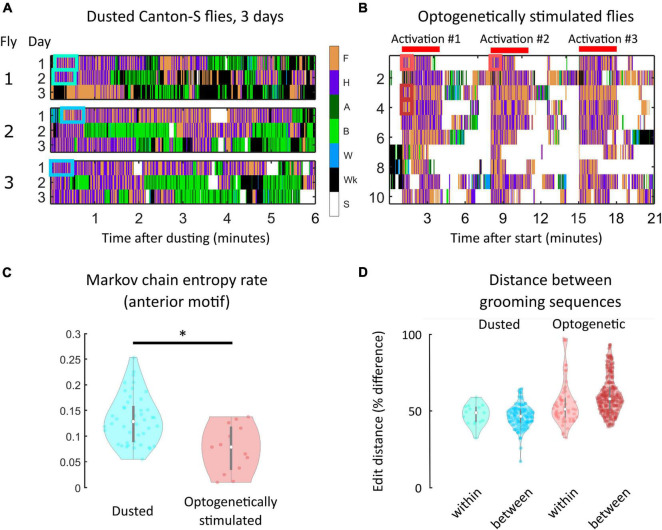
Within-individual grooming differences suggest that non-genetic factors account for a significant portion of variability in behavior. **(A)** Portions of ethograms from three Canton-S flies observed on consecutive days after dust irritant exposure. The differences in ethograms on consecutive days indicate that non-genetic factors must account for some amount of grooming variability. **(B)** Shown are ethograms of 10 Bristle-spGAL4-1 >CsChrimson flies (Zhang et al., 2020). Flies were optogenetically stimulated to induce anterior grooming in three separate 3-min windows, indicated by the red bars. Between these windows, flies still exhibit within-individual grooming variability even though the sensory experience is more uniform than repeated dust exposure. **(C)** Markov chain entropy, a measure of grooming stereotypy, was calculated from anterior grooming syntax. Optogenetically stimulated flies (right) exhibited lower entropies, corresponding to a higher degree of stereotypy, than dusted flies (left). However, optogenetically stimulated flies still exhibited differences in stereotypy between stimulation windows, implicating sources of grooming variability beyond genetic and sensory influences (Supplementary Figure 19). **(D)** To assess grooming stereotypy, edit distance between anterior motif repeats was computed. For dusted within-fly comparisons, we computed the edit distance between the first continuous anterior motif sequence lasting 30 s on consecutive days (light blue). For between-fly comparisons, we computed the edit distance between the first continuous anterior motif sequence lasting 30 s on the first day of experiments (dark blue). For all optogentically-stimulated flies, we computed two similar comparisons: within-session [i.e., comparing the sequences labeled “Activation #1” and “Activation #2” in panel **(B)**; light red] and between-fly (i.e., “Activation #1” for each fly; dark red). For each comparison listed, the median edit distance computed corresponded to around 50% of the sequence length, demonstrating the low degree of stereotypy present in grooming sequences. *significantly different at *p* < 0.05.

Finally, we analyzed dust-induced grooming in 15 Canton-S flies that were assayed on three consecutive days. Since a given individual’s genome remains constant through the three trials, we could isolate the magnitude of grooming variability that is due to differences in sensory experience (since the dusting protocol does not allow for perfect replication of sensory experience) and life history (since flies will have been exposed to the same irritant several times by the end of the experiment). Ethograms from three example flies are provided in Figure 4A (full ethograms are shown in Supplementary Figure 16).

Flies exhibited some longitudinal grooming trends, as the total amount of grooming decreased between the first and third days of the experiment. However, the time to completion of 50% of their total grooming did not decrease, suggesting that flies are not simply grooming quicker, but rather are grooming less consistently over time (i.e., punctuating grooming bouts with more walking and standing) (Supplementary Figure 17). Importantly, intra-individual variability in syntax across three sessions was of the same magnitude as inter-individual variation in syntax (Supplementary Figures 18, 19); that is, flies were no more similar to themselves over time than they were to other flies on a given day. This suggests that non-genetic factors account for a significant proportion of grooming variability.

### Standardizing Sensory Experience Does Not Abolish Grooming Behavioral Variability

To probe the sensory contribution to within-individual variability, we used optogenetic stimulation to induce anterior grooming. 20 Bristle-spGAL4-1 > UAS-CsChrimson flies were tested (Zhang et al., 2020). Figure 4B provides ethograms from this experiment, with red bars indicating the three stimulation windows. Even when sensory experience was controlled in this way, flies exhibited variability in their grooming response.

Grooming stereotypy was again quantified using the entropy rate of the grooming syntax. The entropy rate for optogenetically-stimulated flies was lower than for dusted flies (*p* < 0.05, Wilcoxon rank-sum test), indicating a higher degree of stereotypy in grooming (Figure 4C). We nonetheless observed within-individual variability between stimulation windows, indicating that standardization of sensory input does not fully abolish grooming variability. Supplementary Figure 20 quantifies differences in entropy between sessions for three example flies. In addition, optogenetic stimulation resulted in strong anterior motif grooming behavior, rendering all flies’ transition probabilities very similar (Supplementary Figure 21).

Finally, grooming stereotypy was characterized using edit distance between anterior motif repeats (Figure 4D). This metric, used commonly in bioinformatics, describes the difference between two DNA sequences by calculating the minimum number of base pair substitutions, additions, or deletions that would be necessary for the sequences to be identical. Identical sequences would have an edit distance of zero between them, while maximally different sequences would have an edit distance equivalent to the total sequence length (see Supplementary Methods for details).

Since edit distance measures the similarity between two sequences (rather than the underlying rules that may generate the sequences), it provides a much stricter definition of stereotypy than Markov entropy, which we use as a measure of stereotypy earlier in our analysis. In addition, it is most useful as a stereotypy measure when it is possible to identify a synchronizing “start” signal between sequences of interest, which is not present in the previously described experiments, but is present for optogenetically-stimulated flies. Therefore, when comparing flies across recording sessions or sequences from different flies, the use of edit distance helps answer the specific question, “Do flies perform repeated, similar sequences or subsequences?”

For all dusted flies, we calculated the edit distance within flies across consecutive days to assess whether flies possess stereotyped repeats. For these comparisons, we compared the first continuous anterior motif sequence lasting at least 30 s on consecutive days, shown by the blue boxes in Figure 4A. This particular comparison was chosen to standardize the amount of dust present on the fly to the greatest extent possible and the short-term grooming history for each sequence, and to ensure that each sequence was long enough to exhibit stereotypy if it exists. Anterior motifs were chosen because they consist of only two actions with high transition probabilities between them, making these sequences the most likely candidates for exhibiting stereotypy. These comparisons yielded a minimum edit distance corresponding to a 39.6% difference between sequences. A similar calculation was made between flies, using the first continuous anterior motif sequence lasting at least 30 s on the first day of experiments. These comparisons yielded a minimum edit distance corresponding to a 41.6% difference between sequences.

Edit distance calculations were also performed for all optogenetically-stimulated flies. Within-fly comparisons (i.e., comparing the sequences labeled “Activation #1” and “Activation #2,” red boxes in Figure 4B) yielded a minimum edit distance corresponding to a 31.5% difference in sequences. Between-fly comparisons (i.e., “Activation #1” for each fly) yielded a minimum edit distance corresponding to a 42.8% difference in sequences (Figure 4D). Together, the low degree of stereotypy present in grooming sequences within and between both dusted and optogenetically-stimulated flies shows that grooming sequence variability is present even when genetics, sensory input, and behavioral history are controlled to the greatest extent possible within this experimental paradigm.

## Discussion

Here, we analyzed fly grooming behavior in five different *drosophilid* species and four common *melanogaster* stocks to investigate the relationship between genetic heterogeneity and behavioral variability. Large genetic differences (species-level) correspond to identifiable differences in several grooming features, including the rules governing action transitions known as syntax. Within *melanogaster*, stock lines exhibited smaller variation in grooming syntax, as well as differences in overall activity levels. All flies showed variability in the details of the grooming movement sequence, but increased genetic heterogeneity did not correspond to increased behavioral variability. Analysis of 15 Canton-S flies recorded over consecutive days showed that intra-individual and inter-individual comparisons had similar—high—levels of variability. Optogenetically-stimulated flies also exhibited intra-individual variability in grooming behavior, but less. Taken together, these results demonstrate that large genetic differences result in distinguishable grooming phenotypes, but that genetic heterogeneity within a population does not necessarily correspond to an increase in the range of grooming behavior variability.

### Genetic Influences on Behavioral Variation and Variability

Advantageous behavioral phenotypes that are under genetic control can be selected over evolution to produce populations with differing behaviors. Here, we identified significant inter-species variation in grooming syntax, suggesting a genetic basis for group differences in grooming behavior.

Some species differ from *melanogaster* in their propensity to perform anterior grooming actions; the anterior motif actions are significantly less strongly coupled in non-*melanogaster drosophilids*, suggesting that anterior neuronal circuitry or sensory physiology may differ. We also identified differences in grooming behavior between commonly used *melanogaster* stock lines and between male and female Canton-S flies; most of these differences relate to overall activity levels.

Variability itself is a trait that can also be selected for, but is often overlooked (Geiler-Samerotte et al., 2013). At the individual level, randomizing escape trajectories can be beneficial for escaping predators (Wang et al., 2020), and diversity in search paths can be useful when a group is foraging for food. The fate of the passenger pigeons, hunted to death while flocking together, illustrates the dangers of behavioral homogeneity (Murray et al., 2017). The degree of variability in behavior can be selected for as a bet-hedging strategy against unstable environmental conditions (Kain et al., 2015; Krams et al., 2021). Genetic factors contribute to variability in fly visual, olfactory, and locomotor behaviors (Ayroles et al., 2015; Honegger et al., 2019; Linneweber et al., 2020).

The prevalence of variability in *Drosophila* grooming action sequences suggests that non-stereotyped grooming may be advantageous, perhaps for removing diverse distributions or kinds of debris. We examined whether greater genetic heterogeneity within a population corresponded to greater behavioral variability but did not detect any significant impact.

A recent investigation of unstimulated behaviors in different *Drosophila* species detected differences in spontaneous grooming between species and among individuals within a species (Hernández et al., 2020). Using similar methods, they accurately assigned individuals into species categories and assessed variability among individuals. Our findings are complementary: *drosophilid* species show differences in stimulated grooming behaviors as well, suggesting genetic control, but individuals within a species show variability in grooming, indicating that factors other than genes can influence aspects of the behavioral sequence. Hernández et al. (2020) propose that over the long timescales measured in their assay, internal states may explain the observed fluctuation in action transition probabilities. In the shorter timescales we assayed, where flies are responding acutely to dust, we attribute the variability to inherent flexibility in the behavior itself, produced by differences in sensory input and/or intrinsic stochasticity in the neurons or circuits that coordinate the action sequences. These views are not in conflict and together establish that variability in grooming is widespread—potentially even advantageous—with both genetic and non-genetic factors influencing its expression.

Variability also encompasses individuality in animal behavior, typically defined as a trait-like feature that persists stably over several observations. Individuality has been identified in fruit fly turning (Buchanan et al., 2015), mouse roaming behavior (Freund et al., 2013), and bumblebee foraging (Klein et al., 2017), among others (Linneweber et al., 2020; Takagi and Benton, 2020). In both dust-induced and optogenetically-initiated grooming, we did not find evidence for individuality in action sequence patterns at the resolution we analyzed, but this may be because small contributions from individual tendencies are outweighed by the large amount of variability in the behavior as a whole arising from other causes.

### Environmental and Stochastic Influences of Behavioral Variation and Variability

Our analysis of genetic contributions to behavioral variation and variability in the grooming suggests that at the species level, flies show significant differences in the grooming sequence, especially in the syntax of transition probabilities, that allow accurate classification. Differences in behavior between common lab wild-type stocks also support classification, but the accuracy is lower and the effect size of the differences is smaller.

Genetic factors have been implicated in spontaneous (i.e., unstimulated) grooming behavior in *Drosophila melanogaster* (Yanagawa et al., 2020) and in other *drosophilid* species (Hernández et al., 2020). Our results demonstrate that this is true for dust-induced grooming as well. Both spontaneous grooming (Hernández et al., 2020) and dust induced grooming show individual-to-individual variability within a species. The prevalence of sequence flexibility in all species and in controlled experimental conditions suggests that variability itself is a feature of grooming behavior, not a bug. Individuals with overly rigid grooming sequences might not respond as effectively to changing environmental conditions, such as different kinds of debris or the presence of a potential mate or predator.

The causes of grooming variability are still under investigation. Differences in developmental processes such as neural wiring or synaptic connectivity may contribute to behavioral differences between flies, but our experiments show that even individual flies exhibit variability in grooming over repeated trials with dust or optogenetic stimulation. This suggests that non-genetic factors such as sensory stimuli, internal state, previous experience, and circuit noise contribute to the variability we observe in grooming action sequences. The reduction of variability when sensory inputs are optogenetically controlled supports diversity of sensory stimulation as a contributor. The persistence of variability within individuals suggests that intrinsic stochasticity or noise within the neurons or circuits themselves may also play a role, which are possibilities which should be explored further.

## Materials and Methods

### Genetic Stocks

Canton-S, Oregon-R, Berlin-K, w1118, Bristle- spGAL4- 1 (R38B08- AD; R81E10- DBD), and 20XUAS- CsChrimson. mVenus (attp18) stocks were obtained from the Bloomington Stock Center. Isogenic (more accurately, reduced genetic variability) stocks were made by crossing single males to double-balanced stocks and then back-crossing males to the double balancer stock to isolate single second and third chromosomes. Single pairs were mated to reduce variability of X and IV. ∼ 2 independent isogenic lines from each *melanogaster* stock were generated; note that many attempts to isogenize result in lethality, as anecdotally reported by colleagues. Maximum Variability stocks were obtained by crossing each *melanogaster* strain to double balancers and then crossing the progeny together and selecting against the balancers. This allowed combination of chromosomes for all four strains. The progeny were allowed to interbreed for several generations to enable recombination in the females.

*Drosophilid* species stocks were obtained from Tom Turner, UCSB, and the National *Drosophila* Species Stock Center^1^.

### Data Collection and Processing

Grooming was induced and analyzed as described in Seeds et al. (2014) and Zhang et al. (2020). Three chambers were used in fly dusting assay: dusting chamber (24 well Corning tissue culture plate #3524), transfer chamber and recording chamber. Recording chambers were coated with Insect-a-slip (BioQuip Products Cat #2871A) to discourage wall-climbing and cleaned daily. To control potential circadian effects during assays, trials containing flies of different genotypes were interleaved (allowing for near simultaneity of experiments), and assays were run at the same time each day. Dust-induced grooming assays were performed in 21–23°C. 4–7 day old male flies were anesthetized on ice and transferred to the middle four wells of the transfer chamber. Flies were left in the transfer chamber for 15 min to recover. Approximately 5 mg Reactive Yellow 86 dust (Organic Dyestuffs Corporation CAS 61951-86-8) was added into each of the 4 middle wells of dusting chamber. For fly dusting, the transfer chamber was aligned with the dusting chamber. Flies were tapped into the dusting chamber and shaken 10 times. After dusting, flies and dust were transferred back into the transfer chamber.

Transfer chamber was tapped against an empty pipette tip box to remove extra dust. Dusted flies were then immediately tapped into recording chamber for video recording. The entire dusting process was performed in a WS-6 downflow hood. Approximately 10 individuals were recorded for each genotype. 30 Hz videos were recorded for 50,000 frames (27.78 min) with a DALSA Falcon2 color 4 M camera. A white LED ring right was used for illumination.

Optogenetic stimulation protocol is replicated from Zhang et al. (2020). Further details can be found in the Supplementary Methods.

For each set of experimental comparisons (between species, within species, within individual), a single experimenter performed all dusting assays to eliminate experimenter-related differences that may arise. In total, 390 ethograms were recorded. This number includes species data (*N* = 83), *melanogaster* stocks and isogenic lines = 252), additional male/female Canton-S flies (*N* = 31), individual Canton-S flies followed for three sessions (*N* = 45), and optogenetically stimulated flies (*N* = 10).

Videos were processed through the Automated Behavior Recognition System [ABRS, Ravbar et al. (2019)], trained on a classifier using *melanogaster* flies to generate ethograms. Grooming actions were described previously (Seeds et al., 2014; Hampel et al., 2015). Sub-movements of the grooming actions used in this analysis have not yet been rigorously described and may occur on time scales faster than the 30 Hz recording setup can reliably capture, so they were not considered in this work.

Automated behavioral recognition system was used to generate ethograms. Briefly, the raw video frames were pre-processed to generate 3-channel spatiotemporal images (ST images). Features were extracted in three timescales and saved into different channels of ST images: 1. raw frame; 2. difference between two frames; 3. Spectral features extracted from a 0.5 s window. A convolutional network trained by ST images under different light conditions was then used to label the behavior identified in each frame. A different network was trained for classification of each species due to differences in body size and light conditions. All networks achieved >70% validation accuracy within the training protocol, which reserved 20% of frames as test data after training (see Supplementary Figure 22 for table of classifier performances).

Finally, ethograms were denoised to only include grooming actions that persisted for longer than the approximate duration of one complete leg sweep. Here, we used a cutoff of 150 ms, and eliminated any actions shorter than this duration (fewer than 1% of bouts were removed under this criterion).

### Data Analysis

All ethogram features were extracted using custom-written code in MATLAB 2019a. Grooming progression vectors were generated for each fly by calculating the proportion of each action in 10 non-overlapping windows (2.78 min each), yielding a 70-dimensional vector for each fly (10 windows with 7 behavioral proportions). Grooming syntax was defined as the first-order transition probabilities between actions. Syntax for each fly was calculated as described in Mueller et al. (2019).

Bout duration distributions were generated as described in Mueller et al. (2019), using a normalized histogram with 20 bins of equal width for each behavior. Bin width was determined independently for grooming and non-grooming actions, as standing and walking exhibit longer tailed distributions than grooming actions. Thus, duration distribution vectors were 140-dimensional for each fly. Examples of progression, syntax, and duration distribution vectors can be found in the Supplementary Information.

Statistics for comparisons between grooming features were calculated using built-in MATLAB functions. t-SNE, and multinomial logistic regression classification analysis were performed using built-in MATLAB functions (Supplementary Information).

## Data Availability Statement

The original data analyzed in the study are included in the article/Supplementary Material; further inquiries can be directed to the corresponding author/s.

## Author Contributions

JS: experimental design. JS and NZ: data collection. JM: data analysis. JM and JS: manuscript writing. JM, JS, and JC: manuscript editing. JS and JC: funding acquisition. All authors contributed to the article and approved the submitted version.

## Conflict of Interest

The authors declare that the research was conducted in the absence of any commercial or financial relationships that could be construed as a potential conflict of interest.

## Publisher’s Note

All claims expressed in this article are solely those of the authors and do not necessarily represent those of their affiliated organizations, or those of the publisher, the editors and the reviewers. Any product that may be evaluated in this article, or claim that may be made by its manufacturer, is not guaranteed or endorsed by the publisher.

## Footnotes

1
https://www.drosophilaspecies.com/
